# Gut microbial metabolic signatures in diabetes mellitus and potential preventive and therapeutic applications

**DOI:** 10.1080/19490976.2024.2401654

**Published:** 2024-10-18

**Authors:** Enriqueta Garcia-Gutierrez, A. Kate O’Mahony, Reinaldo Sousa Dos Santos, Laura Marroquí, Paul D. Cotter

**Affiliations:** a Food Biosciences Department, Teagasc Food Research Centre, Fermoy, Co. Cork, Ireland; b APC Microbiome Ireland, University College Cork, Co. Cork, Ireland; c VistaMilk SFI Research Centre, Fermoy, Co. Cork, Ireland; d Departamento de Ingeniería Agronómica, Instituto de Biotecnología Vegetal, ETSIA-Universidad Politécnica de Cartagena, Cartagena, Spain; e School of Microbiology, University College Cork, Co. Cork, Ireland; f Instituto de Investigación, Desarrollo e Innovación en Biotecnología Sanitaria de Elche (IDiBE), Universidad Miguel Hernández de Elche, Elche, Spain; g CIBER de Diabetes y Enfermedades Metabólicas Asociadas (CIBERDEM), Instituto de Salud Carlos III, Madrid, Spain

**Keywords:** Type 1 diabetes, type 2 diabetes, gestational diabetes, gut microbiota, gut barrier, gut dysbiosis, short-chain fatty acid, probiotic, fermented foods

## Abstract

Diabetes mellitus can be subdivided into several categories based on origin and clinical characteristics. The most common forms of diabetes are type 1 (T1D), type 2 diabetes (T2D) and gestational diabetes mellitus (GDM). T1D and T2D are chronic diseases affecting around 537 million adults worldwide and it is projected that these numbers will increase by 12% over the next two decades, while GDM affects up to 30% of women during pregnancy, depending on diagnosis methods. These forms of diabetes have varied origins: T1D is an autoimmune disease, while T2D is commonly associated with, but not limited to, certain lifestyle patterns and GDM can result of a combination of genetic predisposition and pregnancy factors. Despite some pathogenic differences among these forms of diabetes, there are some common markers associated with their development. For instance, gut barrier impairment and inflammation associated with an unbalanced gut microbiota and their metabolites may be common factors in diabetes development and progression. Here, we summarize the microbial signatures that have been linked to diabetes, how they are connected to diet and, ultimately, the impact on metabolite profiles resulting from host-gut microbiota-diet interactions. Additionally, we summarize recent advances relating to promising preventive and therapeutic interventions focusing on the targeted modulation of the gut microbiota to alleviate T1D, T2D and GDM.

## 1. Introduction

Diabetes mellitus or simply diabetes is a chronic disease characterized by high levels of glucose in the blood (hyperglycemia), which is associated with increased life-long risks to several systems in the body through microvascular complications. Despite having fundamentally different pathogenesis, both major forms of diabetes, namely type 1 (T1D) and type 2 diabetes (T2D), are characterized by the loss of functional pancreatic β-cells, which are responsible for the production and secretion of insulin, i.e., the main hormone involved in the regulation of the glucose levels in the blood. While T1D onsets involves significant loss of β-cells due to autoimmune attack, T2D results from mild-to-moderate β-cell loss due to metabolic stress.^1^ T2D is also characterized by varying degrees of insulin resistance in peripheral tissues due to variable β-cell loss,^2^ while, in T1D, insulin resistance can be observed in patients with poor glycemic control or due to intensive insulin therapy.^3^ Gestational diabetes mellitus (GDM) is also a complex metabolic disorder that may result from an underlying β-cell dysfunction.^4^ Of note, besides T1D, T2D and GDM, there are more than 50 subcategories of diabetes described, including monogenic forms of diabetes and the latent autoimmune diabetes of adults (LADA), highlighting the heterogeneity of etiology and presentations of this metabolic condition.^5^

According to the International Diabetes Federation, diabetes affects approximately 537 million adults between 20–79 years, and it is expected that this number will rise to 643 million by 2030 and 783 million by 2045.^6^ T2D is estimated to account for 90% of overall diabetes cases, with T1D constituting around 5–10%. Alarmingly, both the prevalence and incidence of T1D and T2D have been increasing yearly.^6^ The prevalence of GDM varies worldwide but it is also increasing and now impacts 1–30% of women during pregnancy.^7^ Based on these figures, it is urgent that new strategies to address the development and progression of diabetes are found.

Despite their different etiologies, increasing evidence suggests that T1D, T2D and GDM share common elements with respect to their development and progression. The onset of disease arises from a complex interaction between genetic and environmental factors, where the genetic background may modulate disease susceptibility and environmental factors may act as triggers for disease development.^8,9^ Interestingly, while the relationship between T2D and diet is well established and constitutes one pillar of intervention strategies,^10^ the relationship between T1D and non-genetic environmental factors is yet to be fully understood.^11^ However, given that only 10% of genetically predisposed individuals develop T1D,^12^ increasing prevalence suggests that environmental changes might impact T1D development. Moreover, as in T2D, factors like diet and gut microbiota have been proposed as modulators of T1D pathophysiology.^13,14^ Links between T2D and GDM have also been proposed, as GDM can result from high levels of glucose in the blood, and T2D can be developed as a consequence of GDM. Moreover, both diseases may be linked to preexisting metabolic alterations, such as those associated with pre-diabetes.^7^

In the last couple of decades, it has become increasingly apparent that the gut microbiota has a significant impact on human health. A growing body of evidence indicates that bacteria, archaea, viruses, fungi, and their metabolites, have a direct effect on the human body. Negative impacts on these communities of microorganisms and the metabolites they produce, referred to by some as gut dysbiosis, have been associated with a variety of health conditions, both localized and systemic. The immune system has also been linked to most of these conditions, as imbalances amongst the gut microbiota can be accompanied by increased intestinal permeability and translocation of bacterial products that induce local and systemic inflammation.^15^ Examples of such conditions include metabolic syndrome,^16,17^ irritable bowel syndrome (IBS),^18,19^ inflammatory bowel disease (IBD),^20^ colorectal cancer,^21^ rheumatoid arthritis,^22^ and conditions related to the nervous system, such as autism,^23,24^ Parkinson’s disease,^25^ Alzheimer’s disease,^26^ multiple sclerosis^27^ and depression.^28,29^

Notably, impaired pancreatic function has been associated with gut microbiota dysbiosis. Furthermore, it has been suggested that disruption of a proposed microbiota-pancreas axis could contribute to the development and progression of acute and chronic pancreatitis and pancreatic ductal adenocarcinoma.^30–32^ Moreover, diabetes has also been associated with gut microbiota dysbiosis, which suggests that targeted approaches to modulate the gut microbiota could impact the development and progression of such pancreatic conditions.^33^

Here, we summarize the current knowledge relating to the relationship between the etiology of diabetes and the gut microbiota, with an emphasis on gut microbiota-associated metabolites. We also discuss the potential for the use of different strategies, focusing on targeted modulation of the gut microbiota, as a prevention and therapeutic options in these metabolic conditions.

## 2. The pancreatic function and diabetes aetiology/pathogenesis

The pancreas is a glandular organ located behind the stomach in the abdominal cavity, divided into head, body and tail, and has a lobular structure that includes numerous secretory vesicles.^34^ It can be histologically and functionally divided into the exocrine and endocrine pancreas. The former consists of acinar and ductal cells that are involved in the production and secretion of digestive enzymes, while the latter is formed by an endocrine secretory tissue known as the islets of Langerhans or simply pancreatic islets. These structures are comprised of several endocrine cells, namely α-, β-, δ-, pancreatic polypeptide, and ε-cells, which produce and secrete several hormones involved in blood glucose regulation.^35,36^ The two main hormones involved in the control of glucose homeostasis are insulin and glucagon, produced and secreted by β- and α-cells, respectively.^35^ While insulin regulates blood glucose levels by facilitating cellular glucose uptake during postprandial hyperglycemia, glucagon stimulates hepatic glucose release through elevated glycogenolysis and gluconeogenesis and by inhibition of glycogenesis and glycolysis when the glucose levels are low in the bloodstream (e.g., during fasted states).

Numerous articles have extensively reviewed and discussed the etiology and pathogenesis of T1D, T2D, and GDM. Therefore, in the present review, we will just briefly describe the main points involved in the etiology and pathogenesis of these forms of diabetes; we refer readers to the following reviews on the topic.^1,2,7,37–42^

### 2.1. Type 1 diabetes

T1D is an autoimmune disease characterized by the destruction of insulin-producing, pancreatic β-cells. The exact etiology and pathological mechanisms leading to the autoimmune assaults are not fully understood, but it is believed to involve a complex dialogue between the β-cells and the invading immune cells, which culminates in islet inflammation and progressive β-cell dysfunction and death.^43^ This dialogue is mainly determined by a combination of genetic and environmental factors. In genetically predisposed individuals, environmental triggers, such as viral infections or exposure to certain dietary factors, may initiate an autoimmune response, which leads to the activation of immune cells, particularly T cells, that mistakenly target, attack, and destroy β-cells.^44,45^ The continuous destruction of β-cells ultimately results in insulin deficiency, leading to hyperglycemia.^46^ T1D is also associated with the presence of autoantibodies against β-cells antigens, commonly known as islet autoantibodies, that can be detected in the blood before the onset of clinical symptoms and are used as markers for the risk of developing T1D.^44^ In addition to genetics and environmental factors, stochastic events may also play a role in T1D etiology. It has been suggested that the random generation and distribution of T-cell and B-cell receptors as well as epigenetic modifications may be some of the stochastic factors involved in the development of T1D.^47,48^

### 2.2. Type 2 diabetes

T2D pathogenesis involves several mechanisms that lead to impaired insulin secretion and function, including glucolipotoxicity (i.e., excess of glucose and long-chain free fatty acid levels in the plasma), oxidative stress (i.e., excessive production of reactive oxygen species and/or a deficiency in antioxidant defense systems), and endoplasmic reticulum stress (i.e., the endoplasmic reticulum protein folding capacity is overwhelmed). These mechanisms are involved in the development of insulin resistance in splanchnic and peripheral tissues as well as β-cell failure and may contribute to a chronic, low-grade inflammation observed in some tissues (e.g., adipose tissue and pancreas) during T2D progression.^49^

Insulin resistance occurs when insulin-sensitive tissues (e.g., liver, adipose tissue, and skeletal muscle) become progressively less responsive to insulin, resulting in reduced glucose uptake and suppression of endogenous (primarily hepatic) glucose production. When faced with constant hyperglycemia due to impaired insulin action, the body usually increases β-cell mass and β-cell secretory capacity (which is already abnormal at this stage) to compensate for the elevated insulin demand.^50^ Although this compensation may initially maintain normoglycaemia, β-cell function and mass gradually decrease over time due to the constant stresses to which they are submitted (e.g., glucolipotoxicity).^51,52^ Simultaneously, inappropriate secretion and/or responses to glucagon and incretin hormones, such as glucagon-like peptide-1 (GLP-1) and glucose-dependent insulinotropic polypeptide (GIP), may contribute to abnormal glucose levels, particularly in the post-prandial period.^53,54^ It is well known that obesity and lifestyle factors, such as sedentary behavior and high caloric diet, can further exacerbate these mechanisms.^37,38^

### 2.3. Gestational diabetes mellitus

GDM is characterized by any degree of hyperglycemia that is first identified during pregnancy, even though there is a lack of consensus about which glucose levels should be recognized as GDM and treated.^55^ GDM is the most common medical complication during pregnancy and encompasses both cases of undiagnosed T2D identified early in pregnancy as well as true GDM that develops later.^55^ Major risk factors are maternal overweight and obesity, family history of T2D, later age in pregnancy, nonwhite ethnicity and history of giving birth to large infants.^7,56^ GDM is associated with various complications and risks, such an increased risk of developing T2D for both the mother and the fetus later in life.^55,57^ Furthermore, offspring exposed to GDM *in utero* may also be at increased risk for cardiovascular risk factors, obesity, hypertension and dyslipidaemia later in life.^58^

During pregnancy, there is a heightened likelihood for women to release pro-inflammatory cytokines, including but not limited to interleukin 6 (IL-6), tumor necrosis factor-α (TNF-α), interferon-γ a (IFNγ), and C-reactive protein (CRP). This increase in cytokine levels, coupled with elevated levels of placental lactogen, progesterone, and estrogen, can significantly amplify insulin resistance and glucose intolerance.^59^ Additionally, the inflammatory response is linked to hyperglycemia-induced oxidative stress.^60^ This not only leads to various pathophysiological complications but also closely correlates with insulin resistance, causing diminished glucose absorption in peripheral tissues and heightened glucose production in the liver.^61^

## 3. The gut microbiome

Humans are considered to be superorganisms, as we need an associated microbiota to maintain an state of health.^62^ We can find diverse microbial communities in the human body, but those inhabiting the gut, and, more specifically, the colon, are considered the most abundant in number and diversity, accounting for 70% of total numbers (gut microbiota) and approximately eight million genes (metagenome of the gut microbiome).^63^ The gut microbiota comprises bacterial, archaeal, viral and eukaryotic species that co-exist through complex ecological relationships.^64^ While the taxonomic composition of the gut microbiota varies both between and within individuals, there is a level of redundancy in its functional metabolic potential that has been associated with resilience against acute stressors and ecological stability.^65^

### 3.1. Gut microbiota

Amongst the two prokaryote domains present in the gut microbiome, the highest volume of information and level of characterization relates to Bacteria. First communities in the gut become established at birth, influenced in part by delivery mode, and continue to increase in abundance and complexity during the next 1–3 years as milk and solid food are incorporated.^66^ These communities remain relatively stable throughout adulthood, mainly shaped by lifestyle and diet, and their diversity decreases later in life. This loss in microbial diversity has been associated with negative health impacts, and many studies have aimed to characterize the microbiota of humans with above-average lifespans and elucidate the degenerative mechanisms that might have a gut origin. For instance, it has been reported that these centenarians might present a distinctive gut microbiota composition containing certain metabolic pathways that limit the outgrowth of bacterial groups associated with inflammation processes.^67,68^

The main bacterial phyla represented in the human gut are the Bacillota, previously referred to as Firmicutes, in a proportion of approximately 64% under physiological conditions, Bacteroidota (former Bacteroidetes, approx. 23%), Pseudomonadota (former Proteobacteria, aprox. 8%), Actinomycetota (former Actinobacteria, aprox. 3%) and Verrucomicrobiota (former Verrucomicrobia, aprox. 3%).^69,70^ Their distribution is not constant across the digestive tract, as they depend on the ecological micro-environments derived from the gut geography. In addition, their abiotic regulators, namely water activity, gas composition, pH and the presence of molecules with antimicrobial activity secreted by the human cells or by other members of the gut microbiota (e.g., bile salts or antimicrobial peptides), also modulate their distribution.^71^ Thus, the microbiota of the small intestine is dominated by facultative anaerobes capable of growing in the presence of oxygen and bile salts, like some Bacillota and, more specifically, lactobacilli, and Pseudomonadota. Longitudinal progression through the intestine sees a reduction in oxygen levels, accompanied by an increase in bacterial density and diversity. Finally, in the colon, bacterial composition is dominated by anaerobes and bacteria with fermentative metabolism capable of catabolizing substrates reaching the final part of the intestine that have resisted host digestion, such as dietary fibers.^72^

Transversally, we can find heterogeneous communities in the lumen or in the secreted mucus layer attached to the epithelium. This mucus layer is key for the maintenance of gut homeostasis and its structure will determine the bacterial communities inhabiting within.^73^ In the small intestine, the mucus layer is strongly attached to the epithelium, while in the colon we can differentiate an inner layer attached to epithelium, denser and less populated, and an outer layer in contact with the lumen, less dense and exhibiting greater bacterial diversity. The characterization of the transversal bacterial communities remains a challenge, as most studies rely on fecal samples. Arguably, the most reliable data for the analyses of these communities come from human colon biopsies, which have previously identified the presence of *Akkermansia muciniphila*, a species within the phylum Verrucomicrobiota. This mucin-degrader bacteria improves gut barrier integrity and is associated with positive health outcomes, the growth of which is promoted by dietary polyphenols and fermented foods.^74^

Bacterial populations are influenced by other groups coexisting in the gut, although elucidation of these relationships and population dynamics is in its infancy.^75,76^ Bacterial population structure has been shown to correlate with altered fungal composition in certain conditions, such as in autism spectrum disorder and cystic fibrosis, where the abundance of *Candida* spp. was increased.^77,78^ *Saccharomyces*, *Malassezia*, and *Candida* are the fungal genera most commonly present in the feces of healthy humans.^79,80^ The viral component of the gut microbiome is receiving increased attention, with the most abundant members including CrAss-like phage, Microviridae, Siphoviridae, Myoviridae and Podoviridae.^81–83^ Archaea, despite being abundant in nature, are not well characterized in the gut, as most extraction methods are based on bacteria and universal primers for archaea do not cover all lineages,^84^ although shotgun sequencing could be an alternative to overcome this limitation. Most of their functions and their role in health and disease remain to be addressed, but some groups, such as the order Methanobacteriales, have been identified as important for population dynamics. This order is considered ubiquitous in the human gut and one particular species, namely *Methanobrevibacter smithii*, is believed to be the dominant methanogen in this ecosystem, with a prevalence of more than 90%.^85^ Functionally, Methanobacteriales decrease partial pressures of H_2_, increasing the energetic efficiency of primary fermenters.^86^ Other archaea of the order Methanomassiliicoccales use methylated amines, such as trimethylamine (TMA), in methane production.^87^

Overall, these microbial groups will produce metabolites that will trigger effects in the human host beyond the gut.^88^ These microbial metabolites include bacterial compounds such as neurotransmitters, endocrine hormones, quorum-sensing molecules, biogenic amines, bile-derived molecules, branched-chained amino acids, vitamins, antimicrobials, short-chain fatty acids (SCFAs), and bacterial components such as lipopolysaccharide (LPS).^89^

### 3.2. The gut barrier

The intestinal mucosa is also known as “gut barrier”. This highly specialized mucosa is involved in the digestion and absorption of nutrients in the intestine as well as in the homeostasis of the gut microenvironment that ensures the coexistence and bidirectional communication with the gut microbiota.^90^ When the gut barrier is impaired, due to, for instance, infection or inflammation, molecules and translocation of microorganisms that usually would not be able to come across it, experience an increased efflux known as “intestinal permeability” or “leaky gut”.^91^ An increased intestinal permeability was originally associated not only with a number of local disorders, including IBD, IBS and celiac disease, but with other systemic conditions.^92,93^

The key element of the gut barrier is the epithelium, where the mucosa gets in contact with the lumen. This epithelium is formed by a single layer of cells that are replaced every 4–5 days and shed into the lumen.^94^ The epithelium is shaped into small protuberances named villi that increase the absorption surface, and invaginations named crypts where the stem cells are located.^92^ These stem cells differentiate into cell types with absorptive or secretory functions. Absorptive cells include microfold cells (or M cells), which are involved in the immune response in the gut, and enterocytes, the most numerous cell type and whose main function is absorbing nutrients.^95^ The secretory cells are enteroendocrine cells, which secrete hormones and enzymes due to the intestinal stimulations; Paneth cells, with antimicrobial and immunomodulatory function in the form of proteins and peptides; goblet cells, responsible for the production of the mucus layer; and tuft cells, less studied but with immunomodulatory functions.^96^

The paracellular integrity of this layer is maintained by different cell structures. Gap junctions, desmosomes and adherent junctions are lateral connections between cells, whereas tight junctions are protein complexes apically located that control the diffusion of water, ions and small compounds and prevent the passage of bigger molecules.^97^ Tight junctions include different transmembrane proteins, such as occludins, claudins, junctional adhesion molecules, tricellulin and intracellular scaffold proteins (e.g., zonula occludens).^98^

Gut barrier integrity is important in the development of some pancreatic disorders. For example, severe acute pancreatitis is associated with gut barrier impairment that ultimately can cause necrosis and infection as well as multiple organ dysfunction syndrome.^99,100^ Gut barrier integrity is strongly influenced by bacterial metabolites, and treatment with antibiotics and probiotics have been reported as a strategy to alleviate the symptoms and progression of acute pancreatitis.^100^

### 3.3. Metabolites of gut bacteria

**Figure 1.**
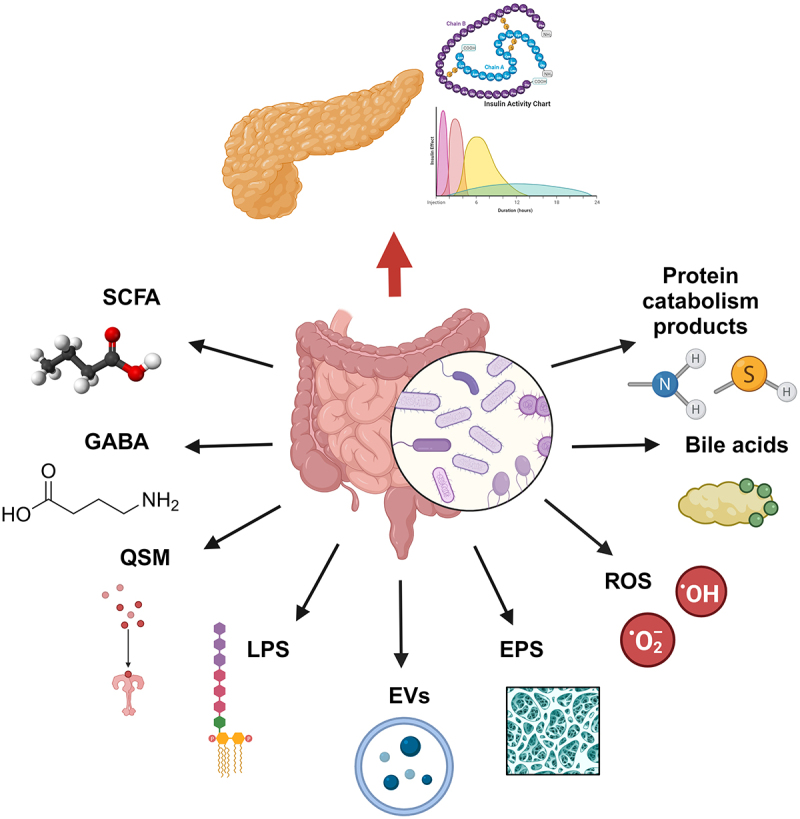
Metabolites produced by gut bacteria that may affect pancreatic functions.

As mentioned above, microorganisms do not exert their action in the gut by direct contact alone. Their metabolism can produce compounds that can have numerous effects on the gut, such as cross-feeding or competition with other members of the microbiota.^101,102^ These metabolites can also interact with the human host in health-promoting or detrimental ways, e.g., vitamins produced by commensals, or toxins produced by enteric pathogens, respectively.^73^ Interkingdom crosstalk has also been observed, wherein certain host receptors are tuned to detect bacterial metabolites such as quorum-sensing molecules, potentially to respond to early-stage infections.^103,104^ This section describes the primary compounds produced by gut bacteria of interest in microbe-microbe and host-microbe interactions affecting pancreatic function (Figure 1).

SCFA – Short chain fatty acids; GABA – gamma-aminobutyric acid; QSM – Quorum sensing molecules; LPS – Lipopolysaccharide; EVs – Extracellular vesicles; EPS – Exopolysaccharides; ROS – Reactive oxygen species. Figure created with Biorender.com.

#### 3.3.1. SCFAs

SCFAs are produced through the fermentation of dietary fiber in the colon. The most abundant are acetate, propionate and butyrate, with formate, valerate and caproate featuring in lower abundance. Acetate and propionate are mainly produced by members of Bacteroidota, while butyrate is primarily synthesized by Bacillota.^105^ SCFAs reduce the pH of the intestinal tract, inhibiting the growth of pathogens. SCFAs also play an important role in the homeostasis of the gut barrier, as they are the main source of energy for the epithelial cells, promoting their proliferation and differentiation and reducing apoptosis.^106^ SCFAs can enhance host defense against enteric pathogens by stimulating the production of antimicrobial peptides by intestinal epithelial cells, including defensins and lysozyme as well as upregulating mucin gene expression.^107^ SCFA can also promote the protein synthesis of tight junctions, more specifically Zo-1 and Occludin, reducing intestinal permeability and strengthening the gut barrier immune function.^99,108^ SCFAs can also disseminate after absorption, with propionate, in particular, being shown to act as a substrate for gluconeogenesis in the liver, highlighting how locally produced metabolites in the gut can have direct systemic effects.^109^

#### 3.3.2. Gamma-aminobutyric acid (GABA)

GABA is the main inhibitory neurotransmitter in the brain that can modulate the gut-brain axis communication.^24,89^ It is produced by the decarboxylation of glutamate by bacteria in the gut, including by species of *Lactobacillus*, *Bifidobacterium* and *Bacteroides*.^110,111^ In the pancreas, GABA is produced and secreted by β-cells at concentrations as high as those found in the central nervous system.^112^ Endogenous, locally secreted GABA is known to exert auto- and paracrine effects as well as metabolic roles in islets cells.^113^ However, besides these endogenous effects, recent evidence suggests that GABA has a strong protective and regenerative effects on the β-cells, especially in the context of massive β-cell loss. Moreover, GABA also improves glucose tolerance and insulin sensitivity, has immunomodulatory and anti-inflammatory effects, and ameliorates diabetes in different models.^114–117^

#### 3.3.3. Quorum-sensing molecules

Quorum-sensing molecules are compounds secreted by bacteria for concentration-dependent transcriptional regulation.^118^ While they primarily coordinate population-level behaviors and cross-species communications, they can also interact with the human host, for example, through crosstalk via G-protein-coupled bitter taste receptors (T2Rs).^119^ As well detecting bitter, sweet and umami flavors in the mouth, T2Rs are also expressed in the gut^120^ and heart and can respond to quorum-sensing molecules from both Gram-negative^121^ and Gram-positive bacteria.^122^ T2Rs can govern inflammatory and oxidative stress responses^123^ and, moreover, secretion of GLP-1, which influences glucose homeostasis. Quorum-sensing molecules also appear to exert an effect on the integrity of the gut barrier. 3-oxo-C12-HSL, a quorum-sensing molecule produced by *Pseudomonas aeruginosa*, provokes apoptosis in macrophages and mast cells, and disrupts tight junctions in the gut *in vitro*.^124,125^ Conversely, other quorum-sensing molecules appear to protect the gut barrier, including the putative host-modified and anti-inflammatory 3-oxo-C12:2-HSL,^126^ indole, and the competence stimulating factor peptide of *Bacillus subtilis*.^127^ The types and concentrations of quorum sensing molecules produced in the gut are likely to be determined by the composition of the microbiota, and more work is required to determine biologically-relevant concentrations and the effect of host polymorphisms on ligand binding.

#### 3.3.4. Extracellular vesicles

Extracellular vesicles (EVs) or membrane vesicles are an end product of secretion from bacteria, which package and export metabolites, possibly mediating cell-to-cell communication, antagonism of competitors, or nutrient sensing.^128^ Recent evidence suggests that some of these EVs could induce insulin resistance and impair glucose metabolism in skeletal muscle, with possible negative implications for the pathogenesis of T2D.^129^ More specifically, EVs derived from *Pseudomonas panacis* impaired the insulin signaling pathway in both skeletal muscle and adipose tissue,^129^ although it is not understood whether effects arise from the vesicles themselves or the molecular cargo within.

#### 3.3.5. Bacterial LPS

LPS is an element of the Gram-negative bacterial outer membrane, which can induce systemic inflammation^130^ and its presence in the blood is considered a biomarker of an impaired gut barrier.^131^ LPS production can also increase reactive oxygen species levels in the gut through activation of host immune cells.^132^ It has been observed that plasma LPS levels were increased in patients with T2D and have been associated with the development of diabetic retinopathy.^130,133^ Moreover, a human longitudinal study, DIABIMMUNE, tracked the development of the gut microbiome from birth until age three years in infants with high-risk genetic HLA haplotypes in Northern Europe, where early-onset T1D is common in Finland and Estonia but is less prevalent in Russia.^134^ The authors characterized the contribution of host-microbe immune interactions to autoimmunity and allergy and, among different observations reported, noted that structurally and functionally distinct LPS could exert different effects with respect to immune stimulation and inflammatory responses, impacting susceptibility to immune diseases such as T1D.^134^

#### 3.3.6. Exopolysaccharides

Exopolysaccharides are a diverse family of macromolecules composed of repeating configurations of monosaccharides. They are key membrane components of many bacteria and can also be secreted, rendering them amenable to purification, and harnessed in the food industry to modulate sensorial profiles of various foods.^135^ Specific exopolysaccharides have been reported to exhibit anti-diabetic properties, such as a heteropolysaccharide from *Lactobacillus plantarum* RJF4, which inhibited α-amylase activity *in vitro*.^136^ Exopolysaccharides from *L. plantarum* YML009 has been shown to mitigate oxidative stress in the gut through scavenging of free radicals.^137^ Additionally, the microbial exopolysaccharides from *Leuconostoc pseudomesenteroides* XG5 delayed T1D onset in non-obese diabetic (NOD) mice, the quintessential murine model for T1D research, through upregulation of GLP-1 secretion, which could be correlated with the increase in butyric acid production in the colon.^138^

#### 3.3.7. Bile acids

Bile acids are produced in the liver from the metabolism of cholesterol. Besides their primary digestive functions, bile acids can also act as signaling molecules and mediate crosstalk between the liver and the gut. Bacteria including *Lactobacillus* sp., *Bifidobacterium* sp., *Enterobacter* sp., *Bacteroides* sp. and *Clostridium sp*. produce bile salt hydrolase enzymes that coordinate the deconjugation of conjugated bile acids, preventing their recycling through the liver and allowing their subsequent modification into secondary bile acids.^139^ Bile acid concentrations and profiles are considered influential in health and disease, with certain bile acids capable of activating host receptors and influencing glucose homeostasis, such as the farnesoid X receptor^140^ and the G-protein-coupled receptor TGR5.^141^ Bile acid sequestrants have also been explored for potential therapeutic applications.^142^ A particularly interesting bile acid, namely tauroursodeoxycholic acid, can increase glucose-stimulated insulin secretion, protect β-cells against cytokine-induced apoptosis, and reduce diabetes incidence in T1D mouse models.^143–145^

#### 3.3.8. Products of protein catabolism

While carbohydrates are the preferred primary energy source, under limiting conditions bacteria can break down proteins into amino acids and peptides that undergo fermentation generating branched-chain amino acids (BCAA), amines, indoles, phenol, hydrogen sulfide and ammonia.^146^ These compounds reduce the uptake, transport, and oxidation of butyric acid by the gut epithelial cells and, ultimately, impact the gut microbiota composition by decreasing the levels of butyrate-producing species, such as *Bifidobacterium* sp., *Blautia* sp. and *Roseburia* sp. This can result in damage to the gut barrier function and a further reduction in butyrate levels by suppression of producer bacteria.

Indole and its derivatives are substances generated through the transformation of tryptophan (Trp), an aromatic essential amino acid that must be acquired through diet, by the gut bacteria. Upon ingestion, a fraction of Trp is used for protein synthesis, while the rest undergoes metabolic pathways within the host organism (e.g. the kynurenine and serotonin pathways) or via intestinal microbes through different metabolic pathways, leading to the production of indole and its derivatives. Indole compounds interact with nuclear receptors, control gut hormones, and regulate the biological effects of bacteria activities.^147^ Many *in vitro* and *in vivo* studies have reported increased expression of tight junctions, reduced intestinal permeability and regulation of proinflammatory cytokine production in the presence of these molecules.^148^ It has been reported that some of these derivatives, such as indolepropionic acid, are associated with lower risk of T2D development,^149^ suggesting promising therapeutic applications and leading to the synthesis of indole analogues with antidiabetic properties.^150^

#### 3.3.9. Betaines

Betaines are a varied group of compounds containing a positively charged nitrogen atom connected to three methyl groups. Some betaines are derived from protein metabolism, while others come from diet, or are by-products of gut microbiota. Trimethylamine (TMA) is an amine compound derived from the transformation of dietary compounds (mainly choline, L-carnitine, and betaine, present in, for instance, eggs, red meats and fish^151^) by few representatives of the gut microbiota that encode the genes for the enzymes responsible for such transformations, like *Acinetobacter* sp and *Pelobacter* sp.^152,153^ TMA is the precursor of trimethylamine N-oxide (TMAO), which has been associated with inflammatory responses resulting in increased cardiovascular disease risk by inducing endothelial dysfunction and affecting the expression of tight junctions.^153^ TMAO has been involved in nephropathy in T2D patients, and it is frequently reported in T2D studies that analyze metabolome.^154^ Recent studies suggest that TMAO could be used as a biomarker for kidney failure progression and mortality outcomes in T2D patients,^155^ and early detection and monitoring could result in better outcomes for patients.^156^ Dietary TMAO regulates the expression of genes related to the insulin signaling pathway, gluconeogenesis, glycogen synthesis, and glucose transport in the liver, which leads to insulin resistance and impaired glucose tolerance in high-fat diet-fed mice.^157^ Additionally, TMAO also increased the expression of the pro-inflammatory cytokine MCP-1 while reducing the mRNA levels of the anti-inflammatory cytokine IL-10, causing adipose tissue inflammation.^157^

Another betaine, namely 5-aminovaleric acid betaine (5-AVAB), a microbial metabolite that can also be found in different foods like milk and meat, has been proposed as a metabolic marker.^158^ Increased serum levels of 5-AVAB were positively associated with worse estimates of obesity, glucose metabolism, and hepatic steatosis after weight loss. Moreover, following weight loss, higher levels of 5-AVAB were independently predictive of adverse alterations in glucose metabolism, suggesting this metabolite could be used for glycemic control.^159^

## 4. The pancreatic microbiota

Outside of infection, the pancreas was once believed to be a sterile organ.^160^ However, this view has been challenged by the recent detection of an associated microbiota using culture-based methods, qPCR, and metataxonomic approaches. Bacteria can be detected in healthy pancreatic samples, which are mainly derived from nonmalignant tissue re-sections or organ donors.^160^ However, rates of detection are consistently higher in samples from patients with pancreatic ductal adenocarcinoma (qPCR detecting 16S rDNA; 15% versus 76%) and members of the phylum *Pseudomonadota* are the most frequently reported.^161^ Pancreatic cyst fluid has also been found to contain a varied and diverse microbiota, particularly formed by *Bacteroides* spp. and *Fusobacterium nucleatum*.^162^ Further investigation is required, especially given the growing consensus regarding the pitfalls and limitations associated with applying sequencing approaches to low-microbial biomass samples, particularly from internal organs.

The origin of pancreatic microbiota remains to be understood. Based on studies where bacterial inocula was administered to mice by oral gavage, it has been hypothesized that bacteria may reach the pancreas from the small intestine and the stomach due to anatomical proximity and reflux-like action of the pancreatic duct.^163,164^ This seems to be a controversial point, as some authors reported an absence of pancreatic colonization after insult to gut barrier integrity. Flow of pancreatic juice and bile in the hepatopancreatic duct can inhibit bacterial migration, while neutral to alkaline pancreatic juice stimulates pH-taxis toward the pancreas and away from the acidic duodenum.^165^ Local immune suppression in the pancreas could prevent immune clearance of bacteria that may translocate from the gut via mesenteric venous or lymphatic drainage.^160,166^ This may signify that underlying pathologies within the pancreas may favor the establishment of bacteria, which would otherwise be prevented in normal physiological conditions.^160,167^ Moreover, the association with, but not causation of, pancreatitis by *Staphylococcus*, *Enterococcus* or *Klebsiella* species seems to implicate the inflammatory environment as being important with respect to facilitating the entry and establishment of these microorganisms in the pancreas.^168,169^ Local pancreatic inflammation has been linked to several chronic conditions, including T1D and T2D.^170,171^ A recent murine study demonstrated that low doses of dextran sulfate sodium, a chemical well known for its effect on disrupting the gut microbiota, decreased butyrate levels in the gut and diminished the expression levels of an antimicrobial peptide in the pancreas that allowed the enrichment of a pathobiont from the family Muribaculaceae in the gut and their translocation to the pancreas. Interestingly, this single pathobiont was enough to trigger local inflammation, β-cell destruction, and the development of insulin-dependent diabetes in germfree mice.^166^ This mechanistic insight may be pivotal in understanding the gut-pancreas axis, but care needs to be taken as such patterns have yet to be revealed by human studies.

Gut bacterial metabolites seem to play a role in the pancreas-gut microbiota bidirectional talk, as highlighted in the aforementioned study through SCFA and antimicrobial peptides. It has also been reported that SCFAs produced by gut bacteria control the production of cathelicidin-related antimicrobial peptide by β-cells, which can convert inflammatory into regulatory immune cells in pancreatic islets. Moreover, cathelicidin-related antimicrobial peptide protected prediabetic NOD mice against autoimmune diabetes.^172^

## 5. Gut microbiota and T1D

Although T1D is considered an autoimmune disease with a strong genetic component, its development has been associated with several environmental factors.^44,45^ It has been recently shown that some of these environmental components can impact gut microbiome composition and its production of butyrate, gut barrier impairment and altered mucosal immunity.^173^

As T1D develops early in life, several human epidemiological studies use cohorts of children to determine the impact of environmental factors on T1D etiology and development. There is some controversy whether being born by C-section is a T1D risk or not, as some studies have reported a correlation between the two elements^174,175^ and others did not.^176,177^ Additionally, there are other elements such as breastfeeding, the use of antibiotics or the timing and mode of exposure to gluten^178^ that are debated to be linked to the establishment and development of the first bacterial communities in the gut of the newborn as the potential triggers of gut barrier disruption and inflammation later on.

**Figure 2.**
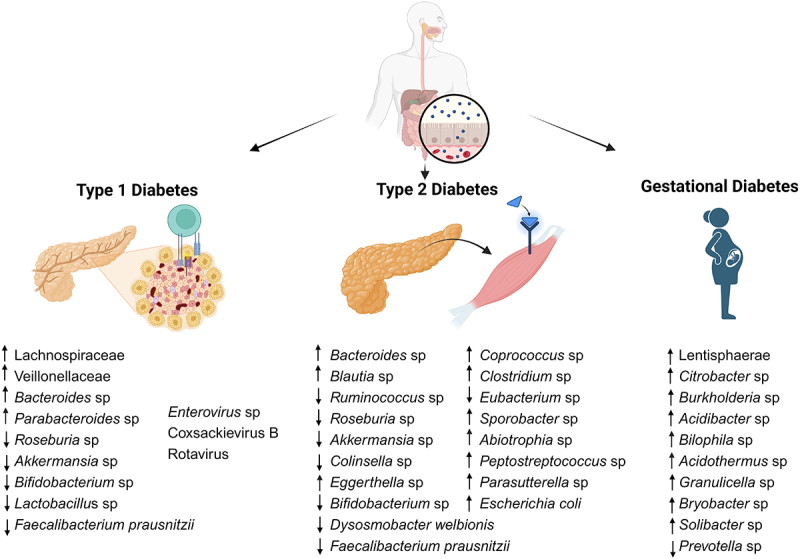
Examples of different gut microbial signatures (family, genus and species levels) positively or negatively correlated with T1D, T2D and GDM at. Viruses in T1D are associated with exposure at earlier stages of development or related to gut dysbiosis. Some common traits, like depletion of SCFA-producing taxa, are correlated with impairment of gut barrier function and pro-inflammatory outcomes, a shared observation in T1D, T2D and GDM. Figure created with biorender.com.

One of the major studies in this vein is The Environmental Determinants of Diabetes in the Young (TEDDY) study, a prospective study that includes clinical research centers in the United States and Europe.^179^ In 2018, the TEDDY study group published two articles based on their investigation of the relationship between the human gut microbiome and the onset of T1D in infants.^180,181^ In the first study, initial results correlated T1D development with the depletion of 11 bacterial genera, including *Lactococcus* sp., *Streptococcus* sp., *Akkermansia* sp. and four unclassified *Ruminococcus* sp., while enrichment of *Parabacteroides* sp. was positively associated with T1D onset (Figure 2).^181^ In the second study, a reduced abundance of the pathways involved in butyrate production was observed in the children that developed islet autoantibodies, along with a higher abundance and diversity of *Streptococcus* sp., with lower relative abundance of *Lactobacillus rhamnosus* and *Bifidobacterium dentium*.^180^ Interestingly, a previous study using the TEDDY population showed that early probiotic supplementation decreased the risk of islet autoimmunity in children at the highest genetic risk of T1D.^182^

The previously referred to DIABIMMUNE study reported that its initial analysis in infants from Finland and Estonia showed a decrease in microbial diversity and a reduction in the number of bacterial genes in children who ultimately developed T1D.^183^ Additionally, they observed a decrease in the families Lachnospiraceae and Veillonellaceae and an increase in the genera *Streptococcus*, *Blautia* and *Ruminococcus*. The metagenome analysis showed a higher prevalence of genes involved in sugar transport and a lower prevalence of the genes involved in amino acid biosynthesis.^183^ A subsequent report identified a distinctive early microbiome in Finnish and Estonian infants when compared to their Russian counterparts. Moreover, they identified several bacteria, such as *Bacteroides* species, that were notably prevalent in Finland and Estonia and lowly abundant in Russians; these bacteria could be producing factors, like LPS, that could potentially suppress the immune system and contribute to the development of T1D. Interestingly, the authors showed that *Bacteroides* LPS was structurally different from *E. coli* LPS and did not reduce the incidence of autoimmune diabetes in NOD mice.^134,134,178^ Finally, a 2018 DIABIMMUNE study showed that *Bifidobacterium infantis*, a bacterium that stimulates β-cell function, is found in only 10% of Finnish infants in this cohort. Moreover, the study suggests that the absence of *B. infantis* could contribute to the prevalence of inflammation-favoring microbes, which could lead to microbiome dysfunction and, ultimately, an increased risk of T1D.^184^

Receptors might play a crucial role in the development of T1D. In studies conducted in NOD mice in a very controlled environment to reduce undesired microbial stimuli, it was found that the myeloid differentiation primary response 88 (MyD88) adaptor protein, used by multiple toll-like receptors, could be critical for T1D development, potentially affecting T cells.^185^ MyD88 signaling could affect gut microbiota composition and function, which in turn modulate the immune responses influencing the development of T1D. Therefore, disrupting MyD88 signaling altered gut microbiota and reduced T1D development. Moreover, normal microbiota was able to alleviate the progression of T1D.^185^

Gut bacteria are not the only potential trigger of T1D development. In fact, several viruses, such as *Enterovirus* sp,^186^ coxsackievirus B,^187,188^ and rotavirus,^189^ have been associated with T1D. It could be hypothesized that an immature and less diverse gut microbiota might provide an environment for these enteroviruses to inflict more damage in the β-cells, or to foster a gut environment that promotes the translocation of microbiota to distal organs, such as the pancreas.

Different observational studies have reported that the gut microbiota from T1D patients is less diverse and stable than the microbiota from healthy subjects.^178^ As for children with prediabetes, they presented a higher relative abundance of *Bacteroides* species and a decreased abundance of *Faecalibacterium prausnitzii*, a bacterium involved in butyrate production.^181^ Other SCFA-producing species, such as *Bifidobacterium adolescentis* and *Roseburia faecis*, are also negatively correlated with the number of autoantibodies,^190^ which are markers of β-cell autoimmunity that strongly associate with T1D development.

## 6. Gut microbiota and T2D

The relationship between gut microbiota and T2D has been studied both in humans and animal models. These studies have reported compositional changes in the gut microbiota profiles, more specifically at phylum and class levels.^191,192^ However, the heterogeneous nature of the available studies, in terms of geography, diet, use of medication, etc., makes it difficult to identify a characteristic microbiota associated with T2D, even at phylum level.^70^ Additionally, it is unlikely that a single species is responsible for the onset of T2D. Some studies have identified higher proportions of opportunistic pathogens, such as *Bacteroides caccae*, *E. coli*, *Clostridium ramosum*, *Clostridium symbiosum* and *Eggerthella lenta*.^70^ Other enriched nonpathogenic genera are *Blautia*, *Coprococcus*, *Sporobacter*, *Abiotrophia*, *Peptostreptococcus*, *Parasutterella* and *Collinsella*.^193^ A few groups of taxa seem to be associated with the early stages and development of T2D in different populations and could potentially be used as biomarkers. Different reports have identified a reduction in the number of butyrate-producing bacteria, such as *Eubacterium rectale*, *F. prausnitzii*, *Roseburia* sp., *Bifidobacterium* sp. and *Ruminococcus* sp., along with lower numbers of the mucin-degrading bacterium *A. municiphila*. It is hypothesized that both *F. prausnitzii* and *A. muciniphila* could offer protection against the development of T2D.^194–196^

A commensal bacterium that has been inversely correlated with T2D in a Japanese cross-sectional study is *Blautia wexlerae*. The genera *Bifidobacterium* and *Blautia* show greater abundance in the Japanese gut microbiome, indicating that gut microbiota representatives vary with geography and cultural differences and have to be taken into consideration.^197,198^ A further study in mice orally administered with *B. wexlerae* showed that there were several metabolites, such as succinate, lactate, acetate, S-adenosylmethionine, acetylcholine and L-ornithine, associated with its action that altered energy metabolism and displayed anti-inflammatory effects under obesogenic conditions (i.e., feeding with high-fat diet); in addition, these metabolites also altered the gut microbiota composition. Altogether, these *B. wexlerae* effects contributed to reducing high-fat diet-induced obesity and diabetes in mice.^198^

Other species have been connected to the development of both obesity and T2D. The commensal gut bacterium *Dysosmobacter welbionis* has been recently associated with prebiotic response, liver health and glucose metabolism in a human study that involved treatment with metformin, an oral biguanide medication used to treat T2D, and prebiotics.^199^ The study found that *D. welbionis* abundance was enriched in the subjects that responded to treatment, being negatively correlated with fasting blood glucose levels. However, metformin did not show a direct effect on *D. welbionis* growth, indicating a complex regulatory connection.^199^ Beyond specific species, other studies have focused on the role played by gut microbiome in nutrient metabolism. A study compared the gut microbiota of 272 T2D against 674 healthy control subjects, finding lower diversity in the T2D subjects, identifying 25 genera that were significantly different and establishing a potential reduction in butyrate production in the T2D cohort.^200^ However, butyrate production was not measured and was predicted using metabolic pathways based on species identification, as the actual metagenome was not studied either, limiting the impact of the conclusions. Many studies support butyrate depletion based on functional prediction from composition,^201^ but establishing actual functionality from gut microbiome and metabolite production would help to understand the connection between gut composition and host metabolism.

Despite a still unclear underlying mechanism, their involvement in insulin resistance and the carbohydrate metabolism of commensals is considered important in T2D.^202,203^ A multi-omics study conducted in 306 individuals, which included a combination of fecal metabolomics, metagenomics and transcriptomics, indicated that fecal carbohydrate metabolites were altered in insulin-resistant patients, particularly monosaccharides fructose, galactose, mannose and xylose, and propionate.^203^ Furthermore, these metabolites were also associated with inflammation, indicating once again the connection between gut microbiota and the immune system.

Glucose homeostasis can be influenced by several bacterial metabolites interacting with G-protein-coupled receptors that are pivotal in the regulation of satiety and digestion. SCFAs can promote the secretion of GLP-1^204^ via binding to GPR43 and GPR119, which are mainly expressed in adipose tissue, the gut, and immune cells.^205^ GLP-1 can modulate satiety in the brain and enhance glucose-stimulated insulin secretion as well as induce insulin gene expression and biosynthesis in β-cells.^204,206^ Bacterial quorum-sensing molecules, specifically acylated homoserine lactones^121^ and autoinducing peptides^207^ can similarly activate the bitter taste receptors T2R38 and T2R14, respectively. SCFAs also enhance glucose uptake by increasing the expression of the glucose transporter type 4.^208^ Propionate is associated with GLP-1-independent enhancement of β-cell function and protection against proinflammatory cytokine- and palmitate-induced islet cell apoptosis.^209^ Importantly, GLP-1 functionality is mediated both by factors influencing its production, such as prebiotics and bile acid chelators,^210,211^ as well as compounds that prolong its half-life, such as dipeptidyl peptidase-4 (DPP4) inhibitors.^212^ DPP4 is an enzyme produced by the gut microbiota that can interfere with the effect of GLP-1 and, along with other isozymes (often associated with *Bacteroides* sp.) can limit the response of certain individuals to drugs to treat T2D such as metformin.^213,214^

Similarly, peptide YY (PYY or peptide tyrosine tyrosine) also influences glucose homeostasis and high levels of PYY are related to insulin sensitivity. PYY is involved in different aspects of gut function, such as delaying gastric emptying and acid secretions, as well as inflammation and cell differentiation. Conversely, it also inhibits glucose-stimulated insulin secretion by pancreatic β-cells.^215^ PYY is produced by the neuroendocrine cells in the ileum and colon and its expression can be regulated by gut bacteria and their metabolites, including SCFA resulting from bacterial fermentation.^216^ Therefore, dietary choices such as consuming high levels of dietary fiber have been associated with the enrichment of fiber-fermenting bacteria such as bifidobacteria and lactobacilli, as well as with higher levels of PPY and GLP-1 levels in plasma.^217^ Gut microbiota dysbiosis may also affect PPY secretion. For instance, antibiotics consumption has been shown to reduce PYY levels, increase enrichment of bacteria associated with obesity and increase food consumption.^218^ Antibiotics-induced gut dysbiosis and its relationship with T2D is, however, not clearly defined and still controversial. While most studies concur on the detrimental impact of antibiotic use on gut microbiota and the onset of T2D, some research indicates that broad-spectrum antibiotics reduce insulin resistance, inflammation, and oxidative stress. Additionally, they lead to an increased abundance of *A. muciniphila*, thereby lowering the incidence of diabetes in mice.^219,220^ Evidence suggests that timing and length of the treatment might be a determining factor in the outcome.^221^ On the other hand, animal models have their intrinsic limitations and, although promising, more work is needed to ensure these therapeutic effects might be similar in humans.

The endocannabinoid (eCB) system is another metabolic pathway involved in glucose homeostasis that is affected by bacterial metabolites in T2D.^222^ The eCB system regulates peripheral glucose and lipid metabolism by influencing the metabolic activities of adipose tissue, the liver, the endocrine pancreas and the gastrointestinal tract.^223,224^ Evidence shows that altered crosstalk between the eCB system and the gut microbiome can result in a variety of health implications such as gastrointestinal, neuroinflammatory and metabolic disorders.^225^ An expanded concept of eCB system includes endocannabinoids (like anandamide (AEA) and 2- arachidonoylglycerol (2-AG) and endocannabinoid-like mediators, like palmitoylethanolamide, which is associated with Trp metabolism in the colon and with protective effects against neuroinflammation.^226^ Supplemented *A. muciniphila* was associated with increased levels of 2-AG and associated acylglycerols, improving gut barrier function and reducing inflammation.^227^ Endocannabinoids have been positively associated with α-diversity and with SCFA-producing bacteria (*Bifidobacterium, Coprococcus*, and *Faecalibacterium*) and butyrate, while negatively associated with *Collinsella*, and the proinflammatory cytokines TNF-ɑ and IL-6. These findings suggesting that SCFA are regulators of the eCB system and partially exert their anti-inflammatory activity via this pathway.^228^

As previously mentioned, BCAAs can impair the gut barrier and reduce the number of butyric acid producers, two risk factors associated with T2D onset.^229^ Amino acid metabolism has been suggested as one of the key predictors of T2D development. More specifically, three BCAAs and three aromatic amino acids were positively associated with T2D: leucine, isoleucine, and valine as BCAAs, and phenylalanine, tyrosine and tryptophan.^229^ High BCAA plasma levels are characteristic of insulin resistance and are correlated with the presence of *Bacteroides vulgatus* and *Prevotella copri*.^230^

Peroxisome Proliferator-Activated Receptors (PPAR) activation has also been linked to T2D, wherein activation of these receptors regulates the transcription of genes involved in inflammation responses; more specifically, *n*-3 fatty acids were reported to promote insulin sensitivity^231^ whereas the TMAO upregulated PPARγ.^232^

## 7. Gut microbiota and GDM

Research indicates substantial alterations in the composition of the gut microbiota in pregnant women. Some taxons have been associated with GDM, namely phyla Bacillota, Bacteroidota, Pseudomonadota, and family Lentisphaerae.^42^ Twenty-seven genera were enriched in GDM, among them seven genera from Pseudomonadota (including *Citrobacter*, *Burkholderia*, *Acidibacter*, and *Bilophila)* and four genera (*Acidothermus*, *Granulicella*, *Bryobacter*, and *Solibacter*) belonging to phylum Acidobacteria, which was positively correlated with glucose blood levels.^42^ Particularly in the last trimester, there is a notable reduction in bacteria crucial for metabolic regulation, accompanied by an increase in Proteobacteria and Actinomycetes, contributing to an inflammatory condition.^233^ Furthermore, the quantity of accumulated fat and stored nutrition is contingent on the gut microbiota condition and composition. Imbalances often result in the formation of easily digestible monosaccharides and activation of lipoprotein lipase through the hydrolysis of undigested polysaccharides, causing excessive storage of hepatic origin substances like triglycerides.^234^ Consequently, dysfunctions in microflora homeostasis of any kind can directly contribute to GDM and disturbances in SCFA levels and composition, leading to disorders in energy metabolism, eating patterns, or blood glucose homeostasis.^60^

Despite having these changes mostly characterized during the second and third trimester of pregnancy, a recent study with 394 pregnant women showed that metabolomic and inflammatory biomarkers associated with developing GDM could be detected during the first trimester.^56^ Specifically, the GDM group showed elevated levels of proinflammatory cytokines (interleukin (IL)-4, IL-6, IL-8, granulocyte-macrophage colony-stimulating factor and tumor necrosis factor-α), and a significant reduction of two branched SCFAs, namely isovalerate and isobutyrate.^56^ Microbiologically, the α-diversity between the GDM and non-GDM individuals was not significantly different, but it was found that *Prevotella* was underrepresented in the GDM group.^56^ An interesting further application of this study was the development of a predictive model using a machine learning approach, which was capable of accurately predict GDM development later in pregnancy based on the studied parameters.

## 8. Gut microbiota and insulin resistance

Insulin resistance is widely recognized as the primary factor underlying the development of different types of diabetes mellitus. Many studies describe the co-occurrence between development of diabetes and changes in gut microbiota composition and gut metabolites. Establishing causality between these changes to identify gut microbiota as the origin of insulin resistance can be more challenging. Animal models have been used to study the development of insulin resistance after receiving gut microbiota from other individuals with diabetes,^56,235^ and the improvement after receiving gut microbiota from healthy phenotype.^236^ This approach identified host changes previously reported in the literature and in the current review, such as loss of species like *A. muciniphila*, impairment of intestinal integrity or increased intestinal permeability associated to development of insulin resistance.^237^ Moreover, the above-discussed gut microbiota metabolites (e.g., LPS, SCFAs, bile acids, BCAAs) have been suggested to be significant contributors to insulin resistance.^238^ However, it has been possible to identify bacterial signatures associated with insulin resistance and insulin sensitivity recently.^239^ These were further associated with a distinct pattern in microbial carbohydrate metabolism and impacting host inflammatory cytokines. Interestingly, the researchers did not find just a group of bacteria but four associations in the patients, including (1) Lachnospiraceae (*Blautia* and *Dorea*), (2) Bacteroidales (*Bacteroides*, *Parabacteroides* and *Alistipes*) and *Faecalibacterium*, (3) Actinobacteria and a non-clustering one (4). These differences also correlated with differences in clinical markers, indicating that there is not a one-answer-fits-all in this area. The study identified species associated with insulin sensitivity (*Alistipes*, and several species from *Bacteroides*, *Bifidobacterium* and *Ruminococcus*). Moreover, their experiments showed that supplementation with species identified in insulin sensitivity (*Alistipes indistinctus*, *Alistipes finegoldii* and *Bacteroides thetaiotaomicron*) improved insulin signalling and resistance in mice, maintaining the pattern of carbohydrate consumption and metabolite production.^240^ This indicates, once more, that is the activity of the given microorganisms what needs to be characterized and not just their presence or absence, therefore needing integrative -omics studies. This study has been of particular importance as it has been able to associate insulin resistance, metabolic syndrome and levels of fecal monosaccharides, and it has attracted a lot of attention.^241^ Their clinical implications involve the potential supplementation of these species promoting insulin sensitivity to reduce insulin resistance.

Despite there seems to be common changes (gut dysbiosis and changes in the gut metabolome that generate gut barrier impairment and inflammation and ultimately insulin resistance and subsequent physiological consequences), a common mechanism in the development of insulin resistance in T1D, T2D and GDM is still unknown. Establishing detailed analyses of common changes and causality would be highly beneficial toward the description of the main pathways and mechanisms underlying the onset and development of diabetes. This information would help to develop therapeutical strategies to target such pathways in an affordable and safe manner, minimizing side effects. However, there is still a lack of consensus in certain aspects, from specific taxa involved in changes to which pathways are involved in the modulation of the immune system via the gut microbiome.^242^ Therefore, more research is needed to clarify these areas.

## 9. Therapeutic options for treating diabetes mellitus based on gut microbiota modulation

Understanding the relationships between the gut microbiota, their metabolites and pancreatic physiology would allow therapeutic interventions targeting the microbial communities in the gut. There are different strategies focusing on modulating the microbiome that have been already applied; others are still under development, but show promising results as a less invasive and more efficient interventions for improving diabetes treatment prospects.^243^ In this section, we provide an overview of some of these methods (Table 1).

**Table 1.** Examples of effects of gut microbiota modulation strategies used in human and animal studies for diabetes control.

	Dietary interventions		Bacterial products	Clinical interventions	
	Diet	Probiotics, prebiotics, synbiotics	Postbiotics Extracellular vesicles	Faecal Microbiota Transplantation	Microbial Transfer Therapy
T1D	Preventive diet: ↓ cumulative incidence of autoantibodies Improvement in gut barrier function ↓ insulitis and delayed T1D onset ↑ Bacteroidota/Bacillota ratio ↑ beneficial serum metabolites, including unsaturated fatty acid and triterpenoids ^244,245^	↓ Islet autoimmunity ↓ HbA_1c_ levels ↑ serum insulin level ^182,246^	↓ FBG ↑ serum insulin level ^247^	↓ FBG ↑ serum insulin level ^248–251^	No results found in Pubmed
T2D	Improvement glycemic control ^252–254^	Improvement glycemic control ↓ levels of circulating LPS ↓ FBG ↓ HbA_1c_ levels ^255–257^	Improvement in gut barrier function Improvement in glucose tolerance ^258^	Improvement blood glucose Improvement insulin sensitivity ↓ HbA_1c_ ^259^	No results found in Pubmed
GDM	Improvement glycemic control ^42^	↓ Plasma glucose levels ↓ Risk of elevated plasma glucose levels ↓ Frequency of pathological results in the glucose tolerance test ↓ Insulin concentration ^260^	No results found in Pubmed	No results found in Pubmed	No results found in Pubmed

HbA1c: glycated hemoglobin. FBG: Fasting Blood Glucose. NOD mice: nonobese diabetic mice. SCFA: short-chain fatty acids.

### 9.1. Dietary interventions

#### 9.1.1. Diet

The structure and composition of the gut microbiota are mainly shaped by birth mode, lifestyle and dietary habits. In the case of T1D, dietary interventions have been conducted at early stages with preventive objectives. FINDIA, a double-blind clinical trial conducted in Finland, studied the impact of bovine insulin-free cow´s milk formula, a whey-based hydrolyzed formula and a whey-based formula from the study group that had the bovine insulin removed.^244^ The study showed that the intake of bovine-free formula during the first 6 months was associated with a reduction in the incidence of islet cell autoantibodies by age three. Moreover, the children who developed autoantibodies showed an increased abundance of *Bacteroides* and a decreased abundance of *Bifidobacterium*.

Other studies that focused on the effect of gluten-free diet in improving the insulin response did not reach conclusive outcomes, as some interventions showed improvement while others did not.^178^ The removal of gluten from the diet was associated with enhanced gut barrier function, a reduction of the inflammation parameters and an improvement in insulin response, although it did not reduce the number of islet cell autoantibodies.^178,261,262^

There is increasing evidence that certain dietary styles, like the Western diet, which is characterized by highly processed foods and fats, might be associated with poor health outcomes. The study of the gut microbiota linked to this diet has highlighted the enrichment of taxonomic groups associated with inflammation.^263^ On the other hand, the consumption of foods containing high dietary fibers and polyphenols, such as the Mediterranean diet, has been associated with the presence of bacterial groups related to lower biomarkers of inflammation and frailty due to the production of SCFAs. Moreover, the consumption of these foods has been reported to improve the gut barrier function^264,265^ as well as postprandial glucose metabolism and insulin sensitivity.^266^ Other studies found that patients receiving a diet with a high content of fiber showed augmented levels of SCFA-producing bacteria, reduced glycated hemoglobin (HbA1c), and increased GLP-1 levels.^267,268^ A recent study showed that supplementing NOD mice with extra virgin olive oil resulted in reduced insulitis and delayed T1D onset. In addition, extra virgin olive oil caused a shift in the composition of fecal microbes, elevating the Bacteroidota/Bacillota ratio and fostering the growth of bacteria that produce SCFAs like *Lachnoclostridium* and Ruminococcaceae_UCG-005. Finally, supplementation with extra virgin olive oil led to augmented levels of beneficial serum metabolites, such as unsaturated fatty acids and triterpenoids, which exhibited a positive correlation with the increased SCFA-producing bacteria and a negative correlation with disease indicators.^245^

NOD mice were also used to evaluate the effects of specialized diets designed to release large amounts of acetate and/or butyrate in the colon, individually and in combination.^269^ The acetate-enriched diet decreased the frequency and number of autoreactive diabetogenic T cells and altered B cell differentiation, while butyrate enhanced peripheral T regulatory cells. Interestingly, when acetate- and butyrate- diets were combined, it was found they acted synergistically, suggesting different mechanisms of action. Additionally, improvements in gut barrier integrity and IL-22 and IL-21 cytokine profiles were noted.^269^ In terms of changes in gut microbial taxa, an increased number of *Bacteroides* was noted following consumption of the acetate-enriched diet. Moreover, the acetate-enriched microbiota showed protection against diabetes in mice who received it. Overall, SCFA-enriched diets showed benefits that could translate into potential effective interventions to humans.

Fermented foods have recently attracted attention because of their reported health benefits, such as alleviation and prevention of metabolic disorders, cognitive improvement, or immune enhancement.^270–273^ Many important studies conducted in animal models have described the beneficial effects of dairy and vegetable fermentations on T2D biomarkers.^252,253^ However, clinical trials in humans show moderate improvements in T2D biomarkers (e.g., glycemic control), with yogurt being most consistently associated with protective effects.^254^ To understand the extent of the antidiabetic benefits of fermented foods, it would be important to increase the recruitment numbers and conduct randomized placebo-controlled trials to limit potential bias in the experimental design of the studies.

Obesity and central adiposity are accepted as being involved in T2D development, and dietary interventions should be carefully considered. For example, popular diet programs which restrict carbohydrate intakes and substitute them with protein over time could result in gut barrier damage and related sequelae, due to increasing concentrations of nitrogen compounds and BCAA that will limit the protective effect of butyrate.^274^

The use of herbs as part of the millennial traditional Chinese medicine (also known as botanical medicine or phytomedicine), has been proven effective in modulating the gut microbiota and controlling the onset and progression of T2D. These combinations of medicinal herbs are a rich source of fiber and phytochemical compounds that favor the growth of beneficial bacteria and the production of beneficial metabolites.^275^ Clinical trials have been conducted to assess the effectiveness of these treatments alone or in combination with Western hypoglycemic pharmacology.^276^ Results showed that the use Shenqi Jiangtang granules (a widely-used treatment for T2D composed of ginseng, ginsenosides, *Astragalus*, *Ophiopogon japonicus*, raspberry, trichosanthin, *Rehmannia glutinosa*, poria, medlar, *Alisma*, *Schisandra*, and yam) might not only improve levels of fasting blood glucose, postprandial blood glucose and HbA1c, but also reduced the risk of developing long-term resistance of the islet function in comparison with just using hypoglycemic treatments.^276^ Similar effects were obtained with Jilinda.^277^ Meta-analyses conducted on several clinical trials concluded that higher numbers of participants would be needed to clarify the seemingly contradictory outcomes of different studies. On the other hand, several recent studies show that Shenqi Jiangtang granules and Jilinda boosted the antidiabetic effects when combined with other hypoglycemic compounds.^278–281^ Other studies showed that these changes associated with the use of traditional Chinese medicine were also positively and negatively correlated with changes in the gut microbiota, including increased abundance of SCFA-producing bacteria Bacteroides, *Faecalibacterium*, *Lactobacillus*, *Roseburia*, and *Bifidobacterium*, and with the decline in abundance of some opportunistic pathogenic bacteria such as *Enterococcus* and *Enterobacter*.^282^ An insight into the mechanisms of the mulberry leaf water extract, also traditionally used to alleviate T2D showed that its supplementation reduced the circulating levels of AEA, 2-AG and LPS, improved intestinal permeability and glucose and lipid metabolism imbalances. These changes were correlated with changes in *Acetatifactor*, *Anaerovorax*, *Bilophila*, *Colidextribacter*, *Dubosiella*, *Oscillibacter* and *Rikenella*, among others, involved in the LPS, AEA and/or 2-AG eCB metabolites.^283^ More studies, however, are needed with larger sample sizes and intervention length and strategies to get further consensus in these gut microbiota-associated changes.

Of note, there is less evidence of metataxonomics changes in gut microbiota related to GDM studies. As GDM is considered a transient stage, many interventions involve diet management, but the studies about this subject are mostly observational. Very few studies have been conducted to characterize how the diet impacts the gut microbiota structure. In the case of GDM, short-term diet management was associated with the change in the Bacillota/Bacteroidota.^42^

#### 9.1.2. Prebiotics, probiotics, synbiotics

More targeted dietary interventions are being developed using probiotics, prebiotics or synbiotics. Prebiotics are defined as “a substrate that is selectively utilized by host microorganisms conferring a health benefit”.^284^ Consumption of prebiotics is typically linked to the production of SCFAs that reduce inflammation and improve gut barrier integrity. Initially, most prebiotics were of carbohydrate origin, although the beneficial compound library has since expanded to include polyphenols and polyunsaturated fatty acids. Carbohydrate-based prebiotics are mostly constituted by inulin, fructo-oligosaccharides and galacto-oligosaccharides that are resistant to the enzymatic digestion in the human small intestine and, therefore, do not increase sugar content in the blood.^285^ Yet, they will be degraded by lactobacilli and bifidobacteria in the colon, promoting the growth of these bacteria while supplying SCFAs to colonic cells. Prebiotics are reported to have clinical beneficial outcomes in the control of glycemic index in T2D, leading to reduction of HbA_1c_ and fasting blood glucose levels.^286^ However, inter-individual variation makes it difficult to generalize a population-level recommended dose. On a positive note, the type of fiber does not seem to be determinant for their glycemic control effect, but the amount needs to be higher than 35 g/day.^286–289^

The use of probiotics has also been reported to improve T2D glycemic control associated with an improvement of gut barrier integrity.^290^ Probiotics are defined as “live microorganisms that, when administered in adequate amounts, confer a health benefit on the host”.^291^ Probiotics have been shown to reduce the levels of circulating LPS, fasting blood glucose, insulin resistance, and HbA1c levels.^255,256^ In T1D, a study supplementing probiotics to a cohort of children reported a reduction in islet autoimmunity.^182^ Species included in probiotic treatments were not homogeneously provided but mainly contained *Lactobacillus* and *Bifidobacterium* species.

Multiple studies affirm that probiotic consumption by pregnant women with GDM can effectively manage glycemia and glucose metabolism as well as lower levels of VLDL cholesterol, triglycerides, and inflammatory markers. The underlying mechanisms, however, remain unexplained and warrant further investigation.^59^ Probiotics primarily confer benefits by reinstating proper microflora, normalizing increased intestinal permeability, and regulating the secretion of pro-inflammatory mediators.^234^ Anti-inflammatory probiotic properties and increased production of bacteriocins and SCFAs, such as butyrate, propane, and acetate, influence insulin resistance biomarkers, acting as chemical messengers from the intestinal lumen to the rest of the body to regulate energy metabolism and fat tissue expansiveness.^59,61^ For instance, butyrate, involved in mucus secretion and supporting the regulatory functions of T lymphocytes, fortifies the protective barrier of the intestinal mucosa and dampens inflammatory reactions.^60^ The antioxidant attributes of probiotics likely result from decreased lipid peroxidation, leading to heightened antioxidant levels or interaction with enzymes, such as glutathione s-transferase, glutathione peroxidase, glutathione reductase, superoxide dismutase, and catalase.^234^ Probiotics may safeguard against oxidative stress by secreting peptides, restoring normal intestinal flora, and eliminating oxidizing compounds or preventing their formation in the bowel.^234^

Synbiotics have also been tested as a potential supplement intervention to alleviate diabetes.^290^ Synbiotics are a “mixture, comprising live microorganisms and substrate(s) selectively utilized by host microorganisms, which confers a health benefit on the host”,^292^ like *Lactobacillus* sp. and *Bifidobacterium* sp. A synbiotic containing *Lactobacillus* sp., *Bifidobacterium* sp., *Streptococcus* sp., yeast and oligosaccharide was shown to reduce the abundance of enteric pathogens and improve fasting blood glucose and HbA_1c_ levels.^70^

### 9.2. Bacterial products

Postbiotics, defined as a “preparation of inanimate microorganisms and/or their components that confers a health benefit on the host”,^293^ have also been identified as potential dietary supplements, such as exopolysaccharides, GABA, supernatants or even the inactivated microorganisms, that could alleviate/prevent diabetes.^247^ Most of the research where these compounds have shown beneficial outcomes against T2D biomarkers has been conducted in animal models.^247^ Thus, more human studies are needed in order to translate these results to human populations. Extracellular vesicles are another emerging class of postbiotics which have attracted recent attention as a potential modulator of the gut ecosystem, including in T1D and T2D.^294^ For the moment, only one study has analyzed them in regard to T2D, finding that extracellular vesicles from *A. muciniphila* improve the gut barrier function and glucose tolerance in an HFD-induced T2D animal model.^258^

### 9.3. Clinical interventions

Other interventions targeting the gut microbiota for treating diabetes require a more clinical approach. This is the case of the fecal microbiota transplantation (FMT) and the microbial transfer therapy. FMT involves the transfer of fecal matter containing fecal microbiota from a healthy individual to a patient with sub-optimal gut microbiota,^295^ whereas the microbial transfer therapy is a modified version of the FMT protocol that requires antibiotic treatments and bowel cleansing before the fecal transfer.^296^ This strategy can transfer entire microbial communities and their metabolites and has proven effective in the treatment of *Clostridium difficile* infections, inflammatory bowel disease, inflammatory bowel syndrome, and autism spectrum disorder-associated gastrointestinal and behavioral disorders.^243,297,298^ However, these techniques are associated with certain challenges, such as the requisite screening of donor samples to prevent the transfer of potentially harmful elements and the efficacy of the treatment will depend on the microbiome of the donor.^243^ FMT was successful in stopping T1D progression in a randomized controlled trial by stabilizing β-cells function and modifying plasma metabolite levels.^248^ Moreover, it was reported a reduction of *Prevotella* in the small intestine that was inversely related to residual β-cell function.^248^ Another study reported a reduction in insulin levels after the FMT, but once stabilized, they were higher than before the transplant.^249^ In a 90-day controlled clinical trial with diet and diet + FMT in a T2D cohort, it was reported that both strategies improved blood glucose and lipids levels as well as blood pressure and body mass index. Furthermore, the addition of FMT treatment to diet induced changes more quickly than diet alone.^299^ Changes in the gut microbiota included increased *Bifidobacterium* levels and decreased sulfate-reducing bacteria levels, mainly *Bilophila* and *Desulfovibrio*.^299^ Another FMT study conducted in patients suffering from metabolic syndrome reported improvements in insulin sensitivity and reductions of HbA_1c_, while levels of butyrate-producing gut bacteria, more specifically *Roseburia intestinalis*, increased.^300,301^ Despite these promising results, more studies are required to assess the applicability and scale-up of these interventions before considering them as a regular treatment.

### 9.4. Experimental microbiome modulation procedures with potential applications to treat diabetes

Phage therapy is still in its infancy but already offers promising results in targeting enteric pathogens that are disrupting gut equilibrium, such as *C. difficile* in ulcerative colitis, invasive adherent *E. coli* in Crohn’s disease or *Ruminococcus gnavus*, enriched in inflammatory bowel disease.^302,303^ Phage therapy has been successfully tested to treat antibiotic-resistant infections derived from diabetic wounds.^304,305^ Phages are already being developed as treatments for intestinal diseases, in the form of phage cocktails, phage vaccines to induce specific immune responses or phage-targeted delivery of therapeutic drugs.^82^ The development of experimental phage interventions to treat T2D, however, has only started very recently. It was found that gavage of an MS2-P22 phage cocktail to a mouse model of T2D, with gut dysbiosis induced by high-fat diet and antibiotic use, rebalanced microbial composition by increasing SCFAs-producing bacteria, reducing representatives of opportunistic pathogens and increasing SCFAs production.^306^ Moreover, there was a reduction in the levels of proinflammatory cytokines and an improvement in the gut barrier function, indicating the potential of this strategy to alleviate T2D symptoms.

Similarly, the use of CRISPR-Cas9 systems is being considered as a tool to edit the gut microbiota and remove harmful members involved in inflammation and dysbiosis, or even to control gene expression and modulate the production of metabolites of interest to maintain the gut barrier integrity and improve T1D and T2D prospects.^307^

Other strategies can help to understand the molecular mechanisms underpinning the effect of the gut microbiota metabolites in T1D and T2D. For example, microfluidics systems in the form of organ-on-a-chip can help to study the interactions between gut microbiota, diet components and human host^308^ and be used in the area of personalized medicine.

In summary, understanding the role of the gut microbiota and its metabolites in the interaction between environmental and genetic predisposition in T1D and T2D can help us design intervention strategies and treatments to improve health outcomes.

## 10. Conclusions and future directions

The gut microbiota and its metabolites are important elements of the gut ecosystem and contribute to the homeostasis of the human body via the communication axis with different organs. However, the interaction between the gut microbiota and the immune system is crucial to maintaining this homeostasis and might be key in the development and progression of diabetes. A better understanding of the connection between the gut microbiota and the food will help address the impact that diet has on diabetes etiology and will help design more targeted intervention strategies to prevent the growth of opportunistic pathogens and the ultimate deterioration of the microbial communities and their metabolites in the gut. Moreover, specific strains and compounds that induce or secrete certain molecules could be incorporated as supplements. However, despite the usefulness of the identification of specific species potentially acting as biomarkers, this is not enough. We have observed the abundance of studies using metataxonomics, while still very few of them report whole metagenomics analyses. These leads to many descriptions of taxa with biomarker potential, but a lack of consensus in which are relevant. Some of them, like *A. muciniphila* or *Bacteroides* have been described in many studies, including reports on how they potentially exert their activity, although it is still difficult to narrow these taxa as biomarkers to monitor progression. On the other hand, metagenomics studies could help to unveil which metabolic pathways are enriched at different stages of diabetes development. Moving a step further, transcriptomics analyses would be an informative way of elucidating the specifics mechanisms involved in the trigger and development of the condition. Nevertheless, these studies are still expensive and require extensive resources. While it is true that the metabolomics analyses can help to bridge that gap, we may only obtain reliable correlations while potentially missing important connections.

Many studies conducted in animal models have shown that fermented foods and gut bacterial metabolites, such as postbiotics, have the potential to prevent and alleviate T1D, T2D and GDM conditions. However, these results are not as clear when the studies are conducted in humans. More double-blinded randomized placebo trials and careful experimental design are needed to assess the range of beneficial effects of these strategies, which would also benefit from the metagenomics and metatranscriptomics approaches. Moreover, incorporating Artificial Intelligence in the form of machine learning would boost the analyses of the massive datasets and help to find patterns and define models to predict the early onset of diabetes. Ideally, identifying these early signatures would translate into targeted treatments, potentially even personalized ones, that would minimize side effects. For example, a rationale use of antibiotics to minimize gut microbiota disruption with metabolic effects downstream. Additionally, it is of note that gut microbiota research is mostly driven by a bacteria-focus, while neglecting the role that other groups, such as viruses and yeasts, can play in the whole ecosystem. More studies are needed to understand the ecological relationships among the different biological entities in the human gut and how they ultimately impact the overall metabolic regulation of the human body.
